# An unconscious man with profound drug-induced hypoglycaemia

**DOI:** 10.11613/BM.2020.010802

**Published:** 2019-12-15

**Authors:** Toon Schiemsky, Guy Vundelinckx, Kathleen Croes, Joris Penders, Koen Desmet, Steven Pauwels, Pieter Vermeersch

**Affiliations:** 1 Laboratory Medicine, University Hospitals Leuven; Department of cardiovascular Medicine, University of Leuven, Leuven, Belgium; 2 Anesthesiology, Ziekenhuis Oost-Limburg, Genk, Belgium; 3 Laboratory medicine, AZ Groeninge Hospital, Kortrijk, Belgium; 4 Laboratory medicine, Ziekenhuis Oost-Limburg, Genk, Belgium

**Keywords:** hypoglycaemia, tramadol, insulin, poisoning

## Abstract

**Introduction:**

Hypoglycaemia has been reported as an unusual complication of tramadol use and in a few cases of tramadol poisoning, but the exact mechanism is not known.

**Case description:**

An ambulance crew was dispatched to an unconscious 46-year old man. A glucometer point-of-care measurement revealed a profound hypoglycaemia (1.9 mmol/L). Treatment with intravenous glucose was started and the patient was transported to the hospital. The patient had several episodes of pulseless electrical activity requiring cardiopulmonary resuscitation in the ambulance and upon arrival in the hospital. Despite continuous glucose infusion the hypoglycaemia was difficult to correct during the next few hours and the patient developed hypokalaemia. Further investigation to identify the cause of hypoglycaemia revealed that insulin and C-peptide were inappropriately raised. A toxicological investigation revealed the presence of tramadol and its metabolites in lethal concentrations. Also acetaminophen, ibuprofen and lormetazepam were present. Ethanol screening was negative (< 0.1 g/L) and no sulfonylurea were detected. The patient developed multiple organ failure, but eventually recovered.

**What happened:**

The hypoglycaemia was caused by inappropriate stimulation of insulin secretion in a patient intoxicated with tramadol. The sudden hypokalaemia was caused by a massive intracellular shift of potassium in response to the hyperinsulinemia, triggered by the intravenous administration of glucose.

**Main lesson:**

To our knowledge, we are the first to document a significant rise in endogenous insulin production in a hypoglycaemic patient presenting with tramadol intoxication. Our observation suggests that hyperinsulinemia could be the cause of the hypoglycaemia associated with tramadol use.

## Introduction

Tramadol is a commonly prescribed weak, centrally acting opioid used for the treatment of moderate to severe pain. It has two main metabolites, O-desmethyltramadol and N-desmethyltramadol. The main activity of tramadol is mediated *via* activation of the µ-receptors and can mainly be ascribed to O-desmethyltramadol, which is 2 to 4 times more analgesic than the parent drug. The second mechanism acts by inhibiting the central serotonin and norepinephrine re-uptake, thereby interfering with the central nociceptive signal transmission (*1*). The most commonly described side effects of tramadol use are vomiting, nausea, drowsiness, dizziness, constipation, dry mouth, seizures, respiratory difficulties and cardiovascular problems. Hypoglycaemia has been reported as an unusual complication of tramadol use and in a few cases of tramadol poisoning (*2*-*5*). A recent study of more than 12 million reports from the United States Food and Drug Administration Adverse Event Reporting System found evidence of significant association of hypoglycaemia with tramadol and methadone use, but not with other opioids (*6*). Diabetes mellitus seems to be a risk factor for this side effect, but the mechanism underlying this association is not known.

## Case report

In response to an emergency call, an ambulance crew and an emergency physician were dispatched to an unconscious man. The 46-year old man had a cerebrovascular accident three years ago and a medical history of rheumatoid arthritis treated with leflunomide. The patient was found unconscious (Glasgow Coma Scale 3/15) in his bed and had vomited. Benzodiazepines were found near his bed. The patient was last seen awake 12 hours before by his wife. A point-of-care (POC) measurement with a glucometer (Accu-Chek Performa, Roche Diagnostics, Rotkreuz, Switzerland) revealed a profound hypoglycaemia (1.9 mmol/L). The patient had no prior history of diabetes mellitus.

The patient was intubated and an intravenous infusion with glucose was started to correct the hypoglycaemia. In the ambulance, the patient developed a pulseless electrical activity (PEA). Cardiopulmonary resuscitation (CPR) was started and 1 mg adrenaline was administered intravenously. This was followed by a return of spontaneous circulation. During transportation and upon arrival at the hospital, a number of additional PEA events occurred requiring CPR.

Upon arrival in the hospital, initial POC arterial blood gas measurements (ABL90 FLEX, Radiometer, Copenhagen, Denmark) revealed profound hypoglycaemia (0.8 mmol/L), acidosis, increased lactate and pCO_2_, decreased O_2_ saturation, and a normal ionogram (Table 1). Oxygen was administered *via* an oxygen mask and the glucose infusion was continued. A new POC analysis 50 minutes later revealed that blood glucose had only increased to 3.6 mmol/L and that the patient now had hypokalaemia. A parallel venous blood sample analysed in the core laboratory confirmed these findings and also showed increased aspartate aminotransferase (AST), lactate dehydrogenase (LD), cardiac troponin T high sensitive (cTnT-hs) and renal failure (creatinine 180 mmol/L, estimated GFR (eGFR): 38 mL/min/1.73m^2^) (Table 2). Urine strip analysis showed ketonuria (1+).

**Table 1:** Point-of-care laboratory results during the first four hours

**Laboratory test (unit)**	**At arrival**	**After 50 min**	**After 130 min**	**After 240 min**	**Reference interval**
Sodium (mmol/L)	143	140	143	143	135 – 145
Potassium (mmol/L)	3.7	2.1	2.6	2.3	3.5 – 4.5
Calcium ionized (pH 7.4) (mmol/L)	1.16	1.17	0.98	1.02	1.12 – 1.23
Chloride (mmol/L)	109	109	109	ND	101 – 111
Glucose (mmol/L)	0.8	3.6	3.3	5.8	3.3 – 6.1
Lactate (mmol/L)	11.0	11.1	9.9	9.9	0.4 – 2.0
pH (pH units)	7.05	6.99	7.21	7.21	7.35 – 7.45
pCO_2_ (kPa)	6.8	7.2	6.4	6.7	4.7 – 6.0
pO_2_ (kPa)	8.7	13.6	11.8	8.4	11.3 – 13.8
HCO_3_^-^ (mmol/L)	14	13	19	19	24 – 31
Base excess (mmol/L)	- 17	- 19	- 9	ND	- 2 to + 3
O_2_ saturation (%)	86	95	95	89	97 – 98
Measurements were performed using the ABL 90 FLEX, Radiometer, Copenhagen, Denmark. ND - not determined. pCO_2_ - partial pressure of CO_2_. pO_2_ - partial pressure of O_2._					

**Table 2:** Core laboratory results of a venous blood sample taken one hour after admission to the hospital

**Laboratory test (unit)**	**Result**	**Reference interval**
Glucose (mmol/L)	3.9	4.1 – 5.9
Potassium (mmol/L)	2.2	3.5 – 5.1
AST (U/L)	179	< 40
ALT (U/L)	219	< 41
LD (U/L)	382	135 - 225
High-sensitive cardiac troponin T (ng/L)	262	*≤* 13
Creatinine (µmol/L)	180	62 – 106
eGFR (CKD-EPI) (mL/min/1.73m^2^)	38	90 – 120
Ethanol (g/L)	< 0.1	< 0.1
Insulin (pmol/L)	583.4	17.8 - 173.0
C-peptide (pmol/L)	4.36	0.37 - 1.47
Measurements were performed using the Cobas 6000, Roche Diagnostics, Rotkreuz, Switzerland. ALT - alanine aminotransferase. AST - aspartate aminotransferase. CKD-EPI - Chronic Kidney Disease Epidemiology Collaboration. LD - lactate dehydrogenase.		

Despite the continuous glucose infusion (a total of 18 g was administered during the first three hours), the emergency medicine physician had difficulties correcting the hypoglycaemia after 130 and 240 minutes. The hypokalaemia also persisted over the next three hours (Table 1). The emergency physician requested a urine toxicology screening (performed using gas chromatography-mass spectrometry, GC-MS) which revealed the presence of tramadol (++++), acetaminophen (+++), lormetazepam (++), citalopram (+), ibuprofen (+) and a trace amount salicylic acid. Serum ethanol and salicylic acid were negative, while the serum acetaminophen concentration was 109.5 mg/L (Table 3). The patient’s home medication included tramadol and lormetazepam.

**Table 3:** Serum concentrations of drugs in a venous blood sample taken 1 hour after admission to the hospital

**Laboratory test (unit)**	**Result**	Therapeutic range*	**Analytical method**
Acetaminophen (mg/L)	109.5	10 - 25	Roche Integra 400
Salicylic acid (mg/L)	< 1.35	20 - 200	Roche Integra 400
Tramadol (mg/L)	9.4	0.1 - 1.0	UPLC-DAD
O-desmethyltramadol (mg/L)	1.3	-	UPLC-DAD
N-desmethyltramadol (mg/L)	3.3	-	UPLC-DAD
Ibuprofen (mg/L)	15.5	15 - 30	UPLC-DAD
Citalopram (mg/L)	Not detected	0.05 – 0.11	UPLC-DAD
Lormetazepam (mg/L)	0.092	0.005 - 0.025	LC-MS/MS
Sulfonylurea (glipizide, gliclazide, glibenclamide, glimepiride, gliquidone) (mg/L)	Not detected	-	UPLC-DAD
*Therapeutic ranges are based on reference 12. UPLC-DAD - reversed-phase ultra-performance liquid chromatography with diode array detection. LC-MS/MS - liquid chromatography-tandem mass spectrometry.			

To exclude hypoglycaemia due to exogenous insulin administration, insulin and C-peptide were measured. Both were significantly increased (Table 2), pointing to increased endogenous production. An abdominal CT scan did, however, not show any evidence for an insulinoma. The hyperinsulinemia could also explain the sudden hypokalaemia *via* an intracellular shift of potassium. Other possible causes of an acute intracellular shift including alkalosis, increased β_2_-adrenergic stimulation, chloroquine intoxication, familial hypokalaemia periodic paralysis or thyrotoxic periodic paralysis were ruled out (*7*).

Tramadol and its main metabolites were quantified by reversed-phase ultra-performance liquid chromatography with diode array detection (UPLC-DAD) in a serum sample taken one hour after admission (see Table 3). Also ibuprofen, citalopram, lormetazepam and sulfonylurea were measured quantitatively (Table 3). A second measurement of serum acetaminophen five hours after admission gave a result of 79.0 mg/L, confirming that a peak level had occurred. Alanine aminotransferase (ALT) and AST were only moderately elevated after five hours (231 and 223 U/L) and 20 hours (403 and 388 U/L). After transfer to the intensive care unit, the patient developed a multiple organ dysfunction syndrome. The cTnT-hs increased to 1742 ng/L after six hours and 1127 ng/L after 19 hours. The patient also developed an aspiration pneumonia and acute respiratory distress syndrome. Eight days after admission, the patient had recovered remarkably and could leave the intensive care. The patient later admitted that he intentionally took an overdose. Informed consent was obtained from the patient.

## Discussion

Insulin and C-peptide were both significantly increased in our patient, pointing to increased endogenous production of insulin. When increased endogenous production is observed, the presence of an insulinoma or another tumour producing insulin like substances should be investigated. Furthermore, cortisol or glucagon insufficiency can cause hypoglycaemia. In addition, a relatively large number of drugs can induce hypoglycaemia and intoxication should be excluded. Most of these drugs, but not all, are used to treat diabetes mellitus (*8*). The probability of intoxication and the hypoglycaemic effect due to these drugs depends on availability to the patient, dose and co-administered drugs, and the time after administration. When these more common causes of hypoglycaemia are excluded, other causes of fasting hypoglycaemia such as inborn errors of metabolism (*e.g.* glycogen storage disorders, fatty acid oxidation disorders, gluconeogenesis disorders) should be investigated (*9*).

Drugs can cause hypoglycaemia by stimulating insulin release, reducing insulin clearance or interfering with glucose metabolism (*8*). Because the liver plays a critical role in glycolysis and gluconeogenesis, hypoglycaemia may develop in patients with hepatic failure due to acute drug-induced liver toxicity (*10*). Based on the clinical presentation and toxicology results, tramadol was considered the most likely cause of the severe hypoglycaemia. Of the multiple drugs co-ingested by the patient, tramadol and acetaminophen were the only drugs present in toxic serum concentrations. In this case, an acetaminophen intoxication causing the hypoglycaemia is not expected since hypoglycaemia typically only occurs after 72 - 96 hours in stage III due to liver failure when liver enzymes are markedly elevated (AST and ALT typically > 10,000 U/L). The other drugs found by toxicology screening were also ruled out as cause of the severe hypoglycaemia. Intoxication with citalopram causing hypoglycaemia has only been described in combination with ethanol (*11*). No evidence in the literature supporting lormetazepam or ibuprofen causing profound hypoglycaemia was found. Salicylates have been described to cause hypoglycaemia in case of intoxication (*8*), but the low urinary concentration does not support this hypothesis.

Hypoglycaemia has been described as an unusual side effect of tramadol use occurring within five days after starting tramadol therapy and in a few cases of tramadol poisoning (*2*-*5*). Only one (fatal) case of tramadol poisoning presenting with hypoglycaemia and cardiac arrest has previously been reported with a plasma concentration of 5.2 mg/L (*5*). The tramadol concentration (9.4 mg/L) found in our patient is far above the therapeutic blood concentration (0.1 – 1.0 mg/L) and also exceeds 2.0 mg/L, which is considered lethal (*12*).

Based on animal studies, two hypotheses have been suggested for the hypoglycaemia associated with tramadol use. The first hypothesis, based on a study with mice, suggests a serotoninergic-mediated mechanism based on the increase of serum insulin and subsequent hypoglycaemia after serotonin administration (*13*). The second hypothesis based on a study in diabetic rats suggests that tramadol lowers plasma glucose by binding to opioid µ-receptors, thereby increasing turn-over of glucose and/or decreasing gluconeogenesis (*14*). Our observation of increased serum insulin and C-peptide in a hypoglycaemic patient presenting with tramadol intoxication suggests that hyperinsulinemia due to adverse activation of the beta cells in the pancreas could be the cause of the known association between hypoglycaemia and tramadol. Since multiple drugs were co-ingested, however, a definite causality between tramadol and this hypoglycaemic cannot be proven.

The patient was prescribed tramadol for pain management. While tramadol is generally preferred for moderate pain in patients with chronic kidney disease (CKD) because it is not directly nephrotoxic, tramadol and its metabolite have been reported to accumulate with advanced CKD (eGFR < 30 mL/min/1.73 m^2^) (*15*). The decreased renal function in our patient might therefore have worsened the tramadol intoxication. Tramadol, like other opioids, has a small therapeutic index (*12*). Accidental tramadol intoxication can therefore occur when patients do not adhere to the prescribed dose.

The sudden hypokalaemia was caused by a massive intracellular shift of potassium in response to the hyperinsulinemia, triggered by the intravenous administration of glucose. Insulin causes this shift mainly via direct stimulation of the Na^+^/K^+^- ATPase. The moderate increase of troponin T one hour after admission and further increase to 1742 ng/L after six hours can be explained by myocardial injury due to CPR and acute cardiac ischemia due to circulatory failure as evidenced by a lactate of 11 mmol/L at arrival in the hospital.

To our knowledge, we are the first to document a remarkably raised endogenous insulin production in a hypoglycaemic patient presenting with tramadol intoxication by measuring serum insulin and C-peptide, suggesting that hyperinsulinemia could, at least sometimes, be the cause of the known association between hypoglycaemia and tramadol.

## Conclusion

When dealing with an unresponsive hypoglycaemic patient, this rare but potential life-threatening side effect of tramadol should be considered, and until the precise mechanism of this intoxication is clearly established, both insulin and C-peptide should be measured.
